# Evaluating Treatment Patterns and the Role of Neoadjuvant Chemotherapy in Plasmacytoid Urothelial Carcinoma: Insights from a Combined National and Institutional Series

**DOI:** 10.3390/cancers16173050

**Published:** 2024-09-01

**Authors:** Syed Rahman, Victoria Kong, Michael Jalfon, David Hesse, Joseph Kim, Jonathan L. Wright, Adebowale Adeniran, Peter Humphrey, Darryl T. Martin, Fady Ghali

**Affiliations:** 1 Department of Urology, Yale School of Medicine, New Haven, CT 06519, USAdarryl.martin@yale.edu (D.T.M.);; 2 Yale School of Medicine, New Haven, CT 06519, USA; victoria.kong@yale.edu; 3 Division of Oncology, Department of Medicine, Yale School of Medicine, New Haven, CT 06519, USA; 4 Department of Urology, University of Washington School of Medicine, Seattle, WA 98195, USA; 5 Department of Pathology, Yale School of Medicine, New Haven, CT 06519, USA

**Keywords:** bladder cancer, chemotherapy, cystectomy, plasmacytoid

## Abstract

**Simple Summary:**

Plasmacytoid urothelial carcinoma (PUC) is a rare and aggressive histologic subtype of urothelial carcinoma of the bladder (BC) with high rates of upstaging and metastases. The aim of our study was to characterize treatment patterns and outcomes using a large national database and our institutional experiences, combating the challenge posed by the rarity of this variant. We demonstrated that, despite an improved pT0 rate associated with NAC, there remains an inconclusive overall survival increase. Additionally, PUC demonstrated a high predilection for peritoneal metastasis, further highlighting the need for investigation into more effective and subtype-tailored treatment options.

**Abstract:**

Background: Plasmacytoid urothelial carcinoma (PUC) is a rare histologic subtype of urothelial carcinoma of the bladder (BC). Our objective was to characterize treatment patterns and outcomes of PUC in the NCDB and our recent institutional experience. Methods: The NCDB was queried for localized PUC cases between 2004 and 2020. Patients with PUC from a single institution (Yale School of Medicine) were also incorporated from 2021 onwards to not double-count patients. The primary outcomes were overall survival and treatment trends. Results: A total of 146 patients were included, 123 from NCDB and 23 from Yale. The median overall survival (mOS) was 28 [IQR 7.5, 50.3] months, 23 [IQR 8.4, 46.3] months for the NCDB patients, and 36 [IQR 4.3, 68.1] for the Yale patients. The mOS for patients receiving neoadjuvant chemotherapy (NAC) was 60.0 [28.0, 91.9] vs. 14.8 months [0, 34.3] for patients without NAC, *p* = 0.038, though the benefit was not preserved in a Cox proportional hazard analysis incorporating the clinical stage, receipt of NAC, and age. The peritoneum was the most common site of metastasis (78.3%), followed by the liver and bones. Conclusion: Our findings underscore the formidable challenge posed by PUC, emphasizing its limited response to current therapies. Despite higher pT0 rates with NAC, the OS benefit remains inconclusive, highlighting the need for more effective treatments.

## 1. Introduction

Plasmacytoid urothelial carcinoma (PUC) is a rare and aggressive histologic subtype of urothelial carcinoma of the bladder (BC) [1]. This variant subtype presents at higher stages, has higher rates of upstaging at surgery, and is more chemoresistance. Additionally, this variant subtype is associated with a higher propensity to spread along pelvic fascial planes (rectally, ureterally, and perivesically), likely corresponding to the aggressive nature of the disease and significant upstaging rates at the time of initial surgery. Overall, these factors together limit treatment efficacy and contribute to an aggressive clinical course that warrants further investigation into optimal treatment algorithms [2,3]. Given these challenges, a deeper understanding of the treatment patterns and the therapeutic responses of PUC is essential for developing novel and more effective management strategies.

The current therapeutic strategies for localized PUC have limited efficacy, often failing to achieve optimal oncologic control. Significant rates of upstaging at the time of surgery as well as high positive margin rates have been observed. Additionally, the utilization of NAC has yet to be reliably correlated with improved overall survival for PUC patients, despite some evidence that pathologic complete response rates (pT0) increase as a result [2,4,5]. Many questions remain regarding the appropriate treatment for this challenging clinical entity in regard to surgical intervention, timing, as well as chemosensitivity as a whole. This underscores the need for further research to elucidate effective treatment protocols and improve outcomes for PUC patients, a goal made significantly more challenging by PUC’s low incidence.

Due to the rarity of this malignancy, clinical characterization has been limited to small institutional series. Further information regarding the effectiveness of therapies and treatment response remains highly dependent on the utilization of multi-institutional cohorts for further investigation. This study aims to provide a comprehensive analysis of clinical outcomes and treatment patterns from a generalizable national series combined with our institutional experience. We hypothesize that PUC will be associated with poor outcomes, but NAC will demonstrate a survival advantage in patients that undergo radical cystectomy.

## 2. Methods

### 2.1. Data Source

The National Cancer Database (NCDB) includes approximately 70% of all new cancer diagnoses annually from over 1500 programs participating in the American College of Surgeons Commission. We queried bladder cancer cases in the National Cancer Database from 2004 to 2020 to identify patients with plasmacytoid histologic subtype urinary bladder cancer (International Classification of Diseases for Oncology, 3rd Edition, topography codes C67.0–C67.9) based on the code 8082. We excluded patients with incomplete data regarding chemotherapy, staging, and treatment. Additionally, retrospective analysis identified 23 additional patients with PUC diagnosed at a single tertiary-care center with available treatment data in the years 2021–2024. The final analytic cohort consisted of 23 patients from the single center retrospective cohort and 123 patients from the National Cancer Database. The 23 patients in the retrospective cohort were diagnosed from 2021 and onwards, while all patients in National Cancer Database were diagnosed prior to 2020 to ensure no repeated tabulation of datapoints. We extracted clinical and demographic characteristics, including age, sex, histology, receipt of NAC, type of chemotherapy utilized (chemotherapy regimen data was only available for the single institution cohort, given the limitations of the national database), clinical T/N staging, and pathologic T/N staging.

### 2.2. Study Outcomes

The primary endpoint was to identify treatment patterns and outcomes for patients with plasmacytoid bladder cancer. This included identifying the frequencies of those managed with cystectomy and those receiving neoadjuvant chemotherapy. Secondary endpoints included identifying associations between treatment and overall survival, including various substratifications by the receipt of neoadjuvant chemotherapy and the type of treatment modality utilized.

### 2.3. Statistical Analysis

We characterized clinical and demographic characteristics using descriptive statistics for both the retrospective single institutional cohort and the National Cancer Database. This included treatment patterns and sites of recurrences. A Kaplan–Meier analysis was used to assess the relationships between chemotherapy receipt and survival as well as the relationship between radiation and survival compared to surgery. Additionally, a Cox proportional hazard model was created, incorporating clinical parameters and NAC receipt, to assess the relationship between treatment type and overall survival. All statistical tests were two-sided, with the statistical significance defined as *p* < 0.05. Statistical analyses were conducted using SPSS Statistics software (version 25, International Business Machines Corporation, Armonk, NY, USA).

## 3. Results

### 3.1. National Cancer Database Series

Between 2004 and 2020, a total of 123 patients were identified with a diagnosis of PUC. The patients were 73.2% male and had a median age of 68.9 [65.4, 72.5]. They were predominantly white (91.5%), with 6.9% identifying as black and only 0.8% as Asian. The majority of patients were treated at academic centers (81.3%), yet a significant proportion (18.7%) were treated at either private practices or mixed academic–private practices. Approximately half of the patients were cT2 and 28.4% were cT3 or higher. Only 8.9% had clinical node positive disease. The majority of patients (61.0%) received radical cystectomy, and 30.9% received primary radiation. Only 21.3% of patients nationally received NAC, without information available about the chemotherapy regimen administered. The majority of patients had pT3 (32.5%) and pT4 disease (42.2%). The complete pathologic and clinical outcomes can be seen in Table 1. The median survival for this group was 23 months [8.4, 46.3].

### 3.2. Yale Series

**Figure 1:**
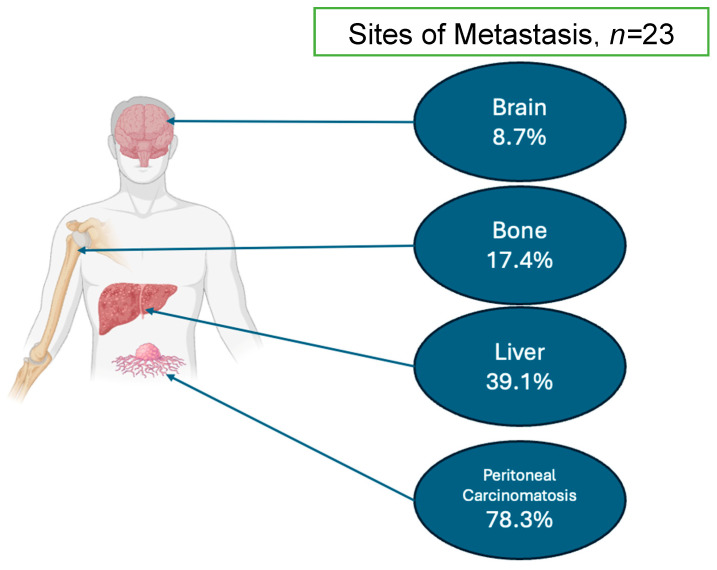
Breakdown of the sites of metastasis identified within the Yale cohort.

Between 2021 and 2024, a total of 23 patients were identified with a diagnosis of PUC. In this institutional series, the patients were 79.2% male and had a median age of 68.4 years [63.0, 75.3]. They were predominantly white (86.9%) and included a higher proportion of black patients compared with the National Cancer Database (13.1%) patients, with 0% identifying as Asian. In this group, 47.2% were cT2 and 22.0% were cT3 or higher. Only 7.3% had clinical node positive disease. The majority of patients (60.8%) received radical cystectomy, and 30.4% received primary radiation. At Yale, 64.2% of patients received NAC, and 92.3% of this was cisplatin-based chemotherapy. Other non-platinum-based regimens administered included therapeutics such as enfortumab vedotin but were rare. The majority of patients had pT3 (61.5%) and pT4 (15.5%) disease. The complete pathologic and clinical outcomes can be seen in Table 1. The sites of metastases (only available in the institutional cohort) are available in Figure 1. The institutional data demonstrated that 78.3% of patients had peritoneal carcinomatosis, 39.1% developed liver metastases, 17.4% developed bone metastasis, and 8.7% developed brain metastases (Figure 1). The median survival for this group was 36 months (CI 4.3–68.1). The comprehensive data on patient demographics, clinical and pathologic staging, treatment patterns, and survival rates for patients in both cohorts are shown in Table 1.

### 3.3. Survival Analysis

**Figure 2:**
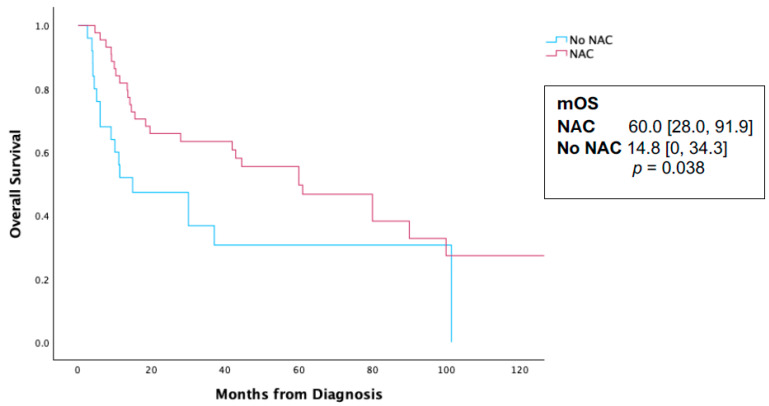
Kaplan—Meier analysis for survival in 146 patients initially diagnosed with localized plasmacytoid urothelial carcinoma and stratified by the receipt of neoadjuvant chemotherapy. NAC—neoadjuvant chemotherapy; mOS—median overall survival.

**Figure 3:**
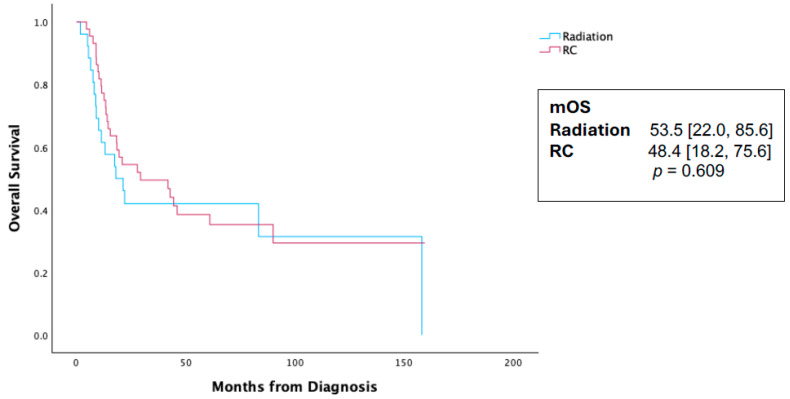
Kaplan–Meier analysis for survival in 146 patients initially diagnosed with localized plasmacytoid urothelial carcinoma and stratified by primary treatment modality. RC—radical cystectomy; mOS—median overall survival.

In an evaluation of the radical cystectomy cohort, pT0 rates for patients who received NAC were 12.3% compared to 0% in patients who did not receive NAC (*p* < 0.001). A Kaplan–Meier analysis (KMA) was performed comparing those who received NAC and those who did not receive NAC in the entire cohort. The NAC receipt was associated with a statistically significant survival difference at 60.0 months [28.0, 91.9] vs. 14.8 [0, 34.3] months, *p* = 0.038 (Figure 2). However, in a Cox proportional hazard regression analysis for survival, the receipt of NAC was not associated with a survival benefit (HR 0.67, CI 0.21–1.61, *p* = 0.26, Table 2). Additionally, primary treatment modality (radiation vs. surgery) was not associated with a survival difference on the KMA (Figure 3) or the Cox proportional hazard regression analysis (HR 0.87, CI 0.64–1.43, *p* = 0.57, Table 2).

## 4. Discussion

Plasmacytoid urothelial carcinoma represents an immensely challenging clinical entity given its high rates of advanced stage at presentation, chemoresistance, and aggressive clinical course. It is particularly difficult to research given the rarity of the malignancy, thus leading to a reliance on several institutional cohorts. We present a large combination series using the National Cancer Database and our recent institutional experience, demonstrating the unique demographics and clinical behavior of PUC, as well as providing information on the efficacy of NAC in improving outcomes for PUC patients. Our study highlights the aggressive natural history as well as the unique pattern of spread associated with PUC.

Patients with PUC experience worse oncologic outcomes compared with the usual urothelial BC patient, as noted by prior groups. Regardless of the receipt of NAC or the treatment modality (primary radiation vs. extirpative surgery), OS is poor [2,4,6,7]. Interestingly, we found approximately 18% of patients with this advanced disease were treated in either private practice settings or in mixed academic–private practices. Nationally, only 13.3% received NAC, compared with 38% in the Yale series, which is similar to the rate reported by Diamantopoulos and colleagues [4]. The MD Anderson group reported that 71% of their PUC patients received NAC [6]. This wide discrepancy in the utilization of NAC in treating PUC may be explained by a lack of consensus amongst providers with respect to the clinical benefits of this regimen, when balanced with the delay of surgery. Patients primarily received cisplatin-based regimens (92.3%), which is consistent with prior reports. This regimen reflects the degree to which PUC care is derived from the clinical evidence of urothelial BC. The majority of patients, both nationally and institutionally, are treated with radical cystectomy (61%), compared with 31% who receive primary radiation. Little is known regarding the efficacy of radiation in treating PUC, yet these findings underline the preference for surgical extirpation as a primary treatment strategy for managing this disease. The role of multimodal treatment protocols remains understudied in variant bladder cancer.

Neoadjuvant cisplatin-based chemotherapy use improves overall survival for urothelial bladder cancer [8], yet there remains a significant lack of consensus regarding its benefit in treating PUC across various institutional cohort series. The University of Washington experience identified no OS benefit with NAC compared with upfront extirpative surgery in their series of 64 patients (26.0 vs. 23.6 months, *p* = 0.3 6) [4]. Similarly, Sood et al. reported no difference with NAC administration in their series of 56 patients (36.4 vs. 33.3% 36-month OS, *p* = 0.61) [6]. A systematic analysis by Kim et al. found no detectable survival benefit associated with NAC, and while pT0 rates did increase with NAC administration, they were significantly lower than those observed in urothelial bladder cancer [2]. In our study, pT0 rate is notably higher with NAC (12.3% compared with 0% without NAC), yet, as has been noted by other groups, many PUC patients will recur despite their complete response, unlike what is observed with urothelial BC [2,8,9]. This likely contributes to why, in a multivariate analysis, NAC as a whole was not associated with a statistically significant survival advantage. Teo et al. reported a series of 81 patients and found NAC was associated with 12% pT0, yet this was not associated with measurable improved survival outcomes [5]. In our series of 143 patients treated across the country, we identified an overall survival benefit with NAC on a univariate analysis (60.0 [28.0, 91.9] vs. 14.8 [0, 34.3] months, *p* = 0.038), though with a wide interquartile range (IQR) and borderline statistical significance. However, this associated survival difference disappears on a Cox proportional hazards regression analysis (HR 0.67, CI 0.21–1.61, *p* = 0.268) when adjusted for the clinical stage, treatment modality, and age. Taken together, these clinical datasets suggest that any survival difference associated with the receipt of neoadjuvant chemotherapy appears inconclusive for PUC.

Patterns of metastasis within the Yale Series are consistent with prior reports, including the recent MD Anderson experience, demonstrating 78.3% of patients with peritoneal carcinomatosis [6,9]. This pattern of spread is distinct from the usual cases of urothelial bladder cancer, yet it has been described in other histologic subtypes of urothelial carcinoma of the bladder, suggesting a distinct metastatic pathway [3,10]. Sood et al. and others have argued that hyperthermic intraperitoneal chemotherapy (HIPEC) may play a role in the treatment of PUC given this pattern of spread. One pilot study of 10 BC patients who received HIPEC at the time of cystectomy demonstrated significant rates of post-operative adverse events, including 43% with small bowel obstruction, 29% with hydroureteronephrosis, 29% with urine leak, and 29% with ascites. A total of 71% of the patients experienced grade ≥ 3 complications [11]. Given the chemoresistance, now well described, of PUC, and the morbidity associated with HIPEC in the setting of the complex reconstruction inherent in bladder cancer surgery, the application of HIPEC in this patient group should be undertaken with extreme caution and thorough assessment of the risks and benefits. It should initially be considered in experimental clinical trials to evaluate proper patient selection. Further questions with regard to HIPEC and intraperitoneal therapy should include the timing of initiation, such as a replacement for adjuvant therapy or as a salvage therapy for peritoneal recurrences like in some mucinous tumors, to determine the most effective treatment protocols. Moreover, careful consideration must be given to patient selection to identify those who are most likely to benefit from these aggressive treatment strategies.

The MD Anderson experience has identified a measurable survival difference for PUC patients treated with systemic checkpoint inhibitors (CPIs, 28.6 vs. 10.0 months, *p* < 0.001) in the salvage setting, suggesting a potential therapeutic benefit from these agents [6]. Interestingly, a recent report of a phase 1b clinical trial of intraperitoneal checkpoint inhibitors (CPIs) has shown relative safety in treating gynecologic cancers, compared with historic HIPEC data, as well as some response. This study evaluated intraperitoneal nivolumab and ipilimumab for recurrent metastatic cervical, ovarian, and uterine cancer, noting an 18.8% objective response rate, and only two patients (8.7%) with grade 3 or higher adverse events. They conclude that this approach is safe and feasible and suggest a recommended phase 2 dose of 3 mg/kg intraperitoneal nivolumab as well as 1 mg/kg ipilimumab [12]. Another early-phase clinical trial at MD Anderson is exploring the safety and efficacy of the intraperitoneal administration of atezolizumab (PD-L1 inhibitor) and bevacizumab (VEGF inhibitor) in advanced recurrent peritoneal mesothelioma refractory to multiple prior lines of therapy. The investigators report this method is able to provide a durable response for a subset of patients with an otherwise grim prognosis, further supporting the potential role of this novel therapy in patients with unmet need. Several clinical case series as well as preclinical models further support investigation of this novel CPI administration method [13,14,15]. Wang and colleagues report the case of a 71-year-old man, diagnosed with pancreatic adenocarcinoma and peritoneal carcinomatosis with refractory malignant ascites, who had failed two lines of systemic chemotherapy as well as seven courses of intraperitoneal chemotherapy (paclitaxel). This patient was given intraperitoneal nivolumab, with a clinical response detected after two weeks and a clinical resolution of his malignant ascites after three months of q2-week instillations [14]. Though promising, these observations and early trials need to be validated in larger prospective studies, yet such clinical experience might serve as a foundation for similar studies in plasmacytoid histology. Thus, combining these clinical observations of the peritoneal pattern of spread, relative chemoresistance, potential response to CPIs, and significant morbidity with HIPEC, one might hypothesize a potential therapeutic role for intraperitoneal CPIs for PUC patients.

Currently, the gold standard treatment option for localized muscle invasive bladder cancer is largely extrapolated from experiences with conventional UC, yet inquiry into the natural history, as well as the molecular underpinnings of PUC, suggests a distinct clinical entity that warrants consideration on its own terms. Genomic and protein expression analysis has highlighted key differences and vulnerabilities between PUC and urothelial BC. For instance, the key defining genomic feature of PUC is the loss of E-cadherin (*CDH1*), a critical cell-to-cell adhesion protein which participates in forming tight cell junctions and whose loss has been described as playing a critical role in the initial dissociation of epithelial cells along a pathway towards metastatic spread [16]. This central genetic event is rare in urothelial BC and may explain the distinct metastatic pattern of spread observed in PUC cases [17,18,19,20,21]. Inquiry into the molecular underpinnings of PUC has also identified markers that may serve as potential therapeutic targets. Some examples include aberrations in well-described oncogenic pathways such as the mammalian target of rapamycin (mTOR), and single-cell-line analyses have found some degree of cytoplasmic phospotase and tensin homolog (PTEN) and phospho-AKT expression in virtually all evaluated PUC samples [22,23,24]. Additional studies demonstrate human epidermal growth factor 2 (HER2) expression in 25–83% of PUC cases [24]. Other targets like nectin-4 and trop-2 have also been observed at high rates among PUC tissue and cited in Hoffman-Censits’ research [25,26]. Studies such as these demonstrate that some level of treatment personalization may help guide treatment decision making in the future. The expression of established therapeutic targets may provide a rationale for future studies involving small molecule inhibitors or antibody–drug conjugates for PUC or treatment with these agents in certain clinical settings [25,27,28].

Further research into the molecular characterization of PUC is also urgently needed. Some data exist in the literature for this group of patients. A small series of 22 patients evaluated using the MSK-IMPACT gene panel has been reported and has identified some similarities and some distinct signatures compared with urothelial bladder cancer [29]. More large-scale studies that might identify molecular subgroups or uncommon genetic vulnerabilities are lacking. Additionally, a thorough evaluation of the tumor microenvironment in PUCs remains incompletely characterized. Studies of the interactions between key immune markers and cells with urothelial BC have been elucidating, laying the groundwork for the development of predictive studies [30,31] and an understanding the potential mechanisms of resistance [29]. Similar studies of PUC are lacking, largely due to the rarity of this malignancy. Questions of this nature will likely require multi-institutional collaborations.

Our study is a retrospective analysis of a combination of datasets from the National Cancer Database as well as our institutional experience, providing a comprehensive overview of the clinical characteristics and outcomes for plasmacytoid urothelial carcinoma (PUC) patients. Several details of therapy are only available for Yale patients, which may limit the generalizability of our findings. Retrospective studies are only correlative, and we draw no causal inference from our findings, highlighting the need for collaborative multi-institutional prospective studies to validate these results. Coding errors and unmeasured confounding are inherent in this study design, which could impact the accuracy and reliability of the data and is difficult to quantify or adjust for. The National Cancer Database is limited in the granularity of its clinical data, particularly regarding specific treatment regimens and metastatic sites of disease. Additionally, the two datasets contributing to our cohort, the National Cancer Database and the Yale series, likely represent heterogenous patient groups, so caution must be undertaken in the interpretation of the combination data. This heterogeneity may introduce variability that could affect the overall outcomes observed. Patients were not double-counted in this series as only Yale patients from non-included National Cancer Database years were evaluated here, ensuring no overlap. Still, there may exist a selection bias with respect to certain unmeasured confounders for patients treated at our single institution, which is suggested by the differences in survival and rates of chemotherapy use. Finally, despite this study’s large sample size relative to prior reports, the number of patients included is rather small, which is a common limitation in studies involving rare cancers like PUC. This challenge is inherent to this rare entity, and thus small correlations may be limited. Additionally, we could not adequately account for many confounders like race, socioeconomic status, and others due to the constraints of our database and the need to avoid over-fitting our analysis. Still, our combined series describes a relatively large and broadly generalizable clinical experience, and thus, despite these limitations, we feel the national and institutional data complement each other and provide valuable information for those treating this difficult disease.

## 5. Conclusions

We present data underscoring the formidable challenge posed by PUC, emphasizing its aggressive clinical behavior and limited response to current therapeutic strategies, including neoadjuvant chemotherapy (NAC). Despite some increases in pT0 rates with NAC, the OS benefit remains marginal, highlighting the need for more effective treatments. Our analysis is limited by its small sample size and retrospective nature, thus small correlations are limited and do not establish causation. Given the distinct metastatic patterns such as peritoneal spread and chemoresistance observed in PUCs, novel approaches are urgently needed and should be further investigated. Future research should focus on innovative strategies through multi-institutional collaboratives and through genomic characterization to improve outcomes for this challenging and underserved patient population.

## Figures and Tables

**Table 1:** Patient and clinical characteristics for both cohorts of patients. NCDB—National Cancer Database; N—sample size; BMI—body mass index; NAC—neoadjuvant chemotherapy; IQR—interquartile range; mos—months.

Variable	Entire Cohort	NCDB	Yale Cohort
N	146	123	23
Male sex	118 (79.3%)	90 (73.2%)	18 (79.2%)
Age	68.8 [IQR 64.2, 73.2]	68.9 [IQR 65.4, 72.5]	68.4 [IQR 63.0, 75.3]
Race			
White	132 (90.4%)	113 (91.5%)	19 (86.9%)
Black	12 (8.2%)	8 (6.9%)	4 (13.1%)
Asian	1 (0.7%)	1 (0.8%)	0%
Other	1 (0.7%)	1 (0.8%)	0%
BMI	N/A	N/A	26.7
Practice type			
Academic	123 (87.0%)	100 (81.3%)	23 (100.0%)
Private	1 (0.6%)	1 (0.8%)	0 (0%)
Mixed	22 (17.5%)	22 (17.9%)	0 (0%)
cT			
0	1 (0.8%)	1 (1.0%)	0 (0%)
1	31 (21.0%)	26 (21.1%)	5 (20.0%)
2	71 (48.3%)	61 (49.5)	10 (47.2%)
3	25 (17.4%)	20 (16.2%)	5 (20.0%)
4	18 (12.5%)	15 (12.2%)	3 (12.0%)
cN+	13 (8.9%)	11 (8.7%)	2 (7.3%)
Treatment			
Radical cystectomy	89 (61.0%)	75 (61.0%)	14 (60.8%)
Observation	12 (8.0%)	10 (8.1%)	2 (8.8%)
Primary radiation	45 (31.0%)	38 (30.9%)	7 (30.4%)
NAC	25 (28.0%)	16 (21.3%)	9 (64.2%)
Agent			
Chemotherapy	N/A	N/A	
Cisplatin-based			21 (92.3%)
Other			
Enfortumab vedotin			2 (7.7%)
pT			
0	18 (12.4%)	14 (11.2%)	4 (15.3%)
1	2 (1.4%)	2 (1.6%)	0 (0%)
2	17 (11.6%)	15 (12.5%)	2 (7.7%)
3	53 (36.3%)	40 (32.5%)	13 (61.5%)
4	56 (38.3%)	52 (42.2%)	4 (15.5%)
pN			
pN0	73 (50.0%)	55 (44.7%)	17 (76.9%)
pN+	42 (28.7%)	37 (30.0%)	5 (23.1%)
pNx	31 (21.3%)	31 (25.3%)	0 (0%)
Median survival (mos)	28 [IQR 7.5, 50.3]	23 [IQR 8.4, 46.3]	36 [IQR 4.3, 68.1]

**Table 2:** Cox proportional hazard analysis for survival in the entire cohort. CI—confidence interval; NAC—neoadjuvant chemotherapy; ref—reference.

	Hazard Ratio (CI)	*p* Value
Clinical T stage (cT1 ref)		
cT2 or more	1.34 (0.97–1.95)	0.08
NAC	0.67 (0.21–1.61)	0.27
Primary radiation (surgery ref)	0.87 (0.64–1.43)	0.57
Age	1.001 (0.996–1.12)	0.46

## Data Availability

Data utilized for this research involves the national cancer database, a database available by the American college of surgeons commission.
